# Economic evaluation of sintilimab plus chemotherapy vs. pembrolizumab plus chemotherapy for the treatment of first-line advanced or metastatic squamous NSCLC

**DOI:** 10.3389/fpubh.2022.956792

**Published:** 2022-08-09

**Authors:** Pingyu Chen, Xintian Wang, Shengwen Zhu, Hongchao Li, Mingjun Rui, Yingcheng Wang, Haikui Sun, Aixia Ma

**Affiliations:** ^1^School of International Pharmaceutical Business, China Pharmaceutical University, Nanjing, China; ^2^Center for Pharmacoeconomics and Outcomes Research, China Pharmaceutical University, Nanjing, China; ^3^Innovent Biologics (Suzhou) Co., Ltd., Suzhou, China

**Keywords:** economic evaluation, NSCLC, PD-1 inhibitors, pembrolizumab, sintilimab

## Abstract

**Background and objective:**

Sintilimab has superior efficacy and safety in patients with advanced or metastatic squamous non-small cell lung cancer (NSCLC), but its cost-effectiveness in China is unclear. This study is to evaluate the cost-effectiveness of sintilimab plus chemotherapy vs. pembrolizumab plus chemotherapy for locally advanced or metastatic squamous NSCLC in China.

**Methods:**

From the perspective of the Chinese health system, the partitioned survival model with three health states was established in a 3-week cycle and a lifetime time horizon. The two-stage method was used to estimate the overall survival hazard ratios to avoid the bias by crossover design in ORIENT-12 and KEYNOTE-407 studies. The anchored matching adjusted indirect comparison method (MAIC) was used for indirect comparison based on the individual patient data from ORIENT-12 and the publicly published KEYNOTE-407 study due to the lack of head-to-head clinical trials. Only direct medical costs were included, and utilities were derived from the published literature in the base case analysis. Sensitivity analysis was also performed to verify the robustness of the model results. In addition, the scenario analysis where the utilities were derived from the Quality of Life Questionnaire-Core 30 (QLQ-C30) scale in the ORIENT-12 by mapping to the EuroQol-5-dimension 5-level (EQ-5D-5L) was carried out to explore the uncertainty of the results.

**Results:**

Compared with pembrolizumab + chemotherapy, sintilimab + chemotherapy incurred a lower lifetime cost ($12,321 vs. 36,371) and yielded fewer quality-adjusted life-years (QALYs) (0.9902 vs. 1.0085), which resulted in an incremental cost-effectiveness ratio (ICER) of $1,314,208/QALY. A sintilimab strategy is a cost-effectiveness option under the WTP of 1–3 times the GDP per capita in China ($11,250/QALY~$33,749/QALY). The utility value of the post-progression, the unit cost of albumin paclitaxel, and the utility value of the progression-free state were the main drivers in the deterministic sensitivity analysis (DSA). According to the probabilistic sensitivity analysis (PSA), sintilimab + chemotherapy was 100% cost-effective when the WTP was 1–3 times China's per capita GDP. The results of the scenario analysis showed that sintilimab + chemotherapy obtained more QALYs (1.2319 vs. 1.1815) and lower costs ($12,321 vs. 36,371), which implied that sintilimab + chemotherapy may dominate the pembrolizumab + chemotherapy.

**Conclusion:**

Compared with pembrolizumab + chemotherapy, sintilimab + chemotherapy is more cost-effective for first-line treatment in Chinese patients with locally advanced or metastatic squamous NSCLC.

## Introduction

Lung cancer has become one of the cancers with the highest morbidity and mortality in the world (1). According to the survey statistics of the International Agency for Research on Cancer in 2018, there were 2,093,876 new lung cancer patients and 1,761,007 new lung cancer deaths worldwide 2018, accounting for 11.6% of new cancer cases and 18.4% of new cancer deaths, respectively. According to the report of the China National Cancer Center, in China, there were 787,000 new cases of lung cancer and 631,000 new lung cancer deaths in 2015, with an incidence rate of 35.96/100,000 (2). Squamous (SQ) non-small cell lung cancer (NSCLC) cases account for about 17% of the total NSCLC cases where the proportion of patients with negative driver gene mutations is about 80% (3). PD-1 drugs provide a choice for the treatment of these patients with negative driver genes. PD-1/L1 is a surface co-inhibitory protein that belongs to the immunoglobulin superfamily (4). By binding with ligands, it can downregulate the immune system response to treat patients. According to the *Guidelines of Chinese Society of Clinical Oncology (CSCO) for Immune Checkpoint Inhibitor Clinical Practice 2021* and the *Guidelines of Chinese Society of Clinical Oncology (CSCO) for Non-Small Cell Lung Cancer*, pembrolizumab combined with chemotherapy was recommended as Class 1A first-level treatments, and sintilimab combined with platinum-based chemotherapy as a Class 1A second-level recommended therapy (5, 6).

Sintilimab is a programmed cell death protein 1 (PD-1) inhibitor that produces a tumor immune response by binding to PD-1, blocking the binding of PD-1 to programmed cell death ligand 1 (PD-L1) and programmed cell death ligand 2 (PD-L2), relieving the immunologic suppression and activating the function of T cells. In June 2021, sintilimab combined with chemotherapy (gemcitabine plus platinum) was approved for the treatment of first-line locally advanced or metastatic squamous NSCLC in China based on the ORIENT-12 study, which was a randomized, double-blind phase III clinical trial conducted in China (7). The study was a head-to-head clinical trial of sintilimab combined with chemotherapy in Chinese patients with advanced or metastatic squamous NSCLC. The main outcomes were published in January 2021, and until the data cutoff date (25 March 2020), the progression-free survival (PFS) of sintilimab combined with the chemotherapy group was significantly better than placebo combined with the chemotherapy group (5.5 vs. 4.9 months).

Although sintilimab combined chemotherapy is recommended in the CSCO guideline 2021, there is no study proving its cost-effectiveness. According to the *China Guidelines for Pharmacoeconomic Evaluations 2020* (8), it is recommended that the selection of a comparator should prioritize standard treatment for the same indication. Pembrolizumab combined with chemotherapy is the standard treatment for patients with advanced or metastatic squamous NSCLC. Although the price of sintilimab is much lower than pembrolizumab, the difference in clinical efficacy and health outcome between the two strategies is unclear due to the lack of head-to-head clinical trials. Therefore, this study aimed to evaluate the cost-effectiveness of sintilimab combined with chemotherapy vs. pembrolizumab combined with chemotherapy for the treatment of first-line advanced or metastatic squamous NSCLC in China.

## Methods

### Model structure

**Figure 1:**
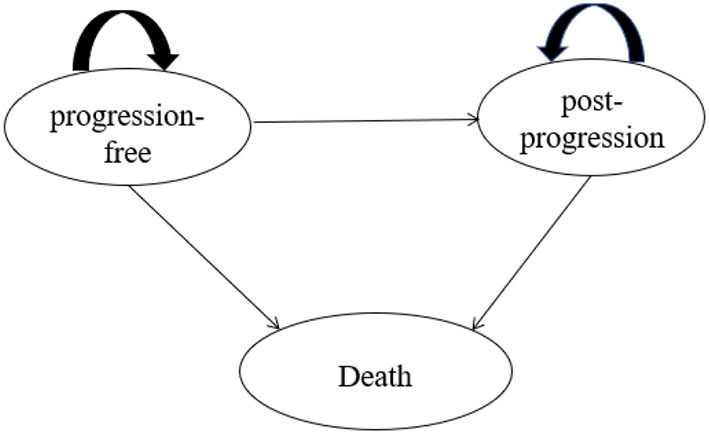
Partitioned survival model structure.

A three-state partitioned survival model (PSM) (9) was developed in Microsoft Excel (Figure 1) to estimate long-term health outcomes and costs for different interventions. One published study proved that the Markov model and PSM would get similar results under the same model structure and assumptions, but it is relatively easier to construct the PSM and more appropriate when individual data are available (10). Additionally, the PSM was also preferred for the economic evaluation of interventions with limited health status according to *NICE DSU Technical Support Document 19* and *China Guidelines for Pharmacoeconomic Evaluations 2020*.

The PSM included three states, namely, progression-free (PF), post-progression (PP), and death. The PF state was defined as the initial state of patients, and patients were assumed to receive treatments until disease progression or death occurred. During the PF state, patients were in a stable state or remission. Patients who experienced disease progression would transfer to the PP state, and the definition of progression was consistent with the Response Evaluation Criteria in Solid Tumors (RECIST) in ORIENT-12. Within the PP state, patients would move on to receive subsequent therapies that were aligned with the trial data of ORIENT-12, and they would experience a lower utility weighting than in the PF state. Patients in PF and PP states both have a certain probability of death.

Each health state in the model is associated with corresponding costs and quality-of-life levels. Quality-adjusted life years (QALYs), life years gained (LYGs), and total costs were measured throughout the lifetime. The cycle length of the model was 3 weeks, which was aligned with the administration cycle of the drugs in the ORIENT-12. Only direct medical costs were taken into consideration since the Chinese healthcare system perspective was adopted. All costs and health outcomes were calculated based on the 2020–2021 prices and discounted at 5% according to the *China Guidelines for Pharmacoeconomic Evaluations 2020*. In addition, 1–3 times GDP per capita in China ($11,250–$33,750 per QALY gained in 2020, US$1 = 6.44 CNY) was considered as a willingness-to-pay threshold for the cost-effective analysis (8).

### Patient population

The target population of the economic evaluation was Chinese patients aged older than 18 with histologically or cytologically confirmed diagnosis of stage III or IV squamous NSCLC who had not previously received systemic treatments. For the sintilimab plus chemotherapy group (intervention group), patients received sintilimab 200 mg every 3 weeks in combination with gemcitabine and either cisplatin or carboplatin for four cycles. Patients without progression after combination therapy would continue to receive sintilimab 200 mg monotherapy as a maintenance treatment for up to 24 months. For pembrolizumab combined with chemotherapy (comparator group), patients received pembrolizumab 200 mg plus carboplatin and paclitaxel/nab-paclitaxel every 3 weeks. After four cycles, patients continued to receive only pembrolizumab every 3 weeks until 24 months. The treatments were consistent with corresponding clinical trials ORIENT-12 and KEYNOTE-407. The detailed information associated with the trial design, efficiency, and safety presented in the ORIENT-12 and KEYNOTE-407 trials can be obtained in the published literature (7, 11).

### Model inputs

#### Efficacy data

Efficacy data for the intervention group was obtained from the ORIENT-12 trial. The individual patient-level data (IPD) of ORIENT-12 was obtained through the company Innovent Biologics (Suzhou) Co., Ltd., Suzhou, Jiangsu, People's Republic of China's official authorization. The efficacy data for the control group was derived from published literature of KEYNOTE-407. Due to the lack of head-to-head clinical trials between the two groups, indirect comparisons are required in this study.

Given the existence of crossover will cause the HR value of OS to be underestimated, the two-stage method (12, 13), which is aimed at reducing the bias, was used for both sintilimab and pembrolizumab groups before indirect comparison. The adjustment results are shown in Table 1.

**Table 1:** HRs for PFS and OS after adjustment of two-stage method.

**Adjustment results**	**HR-OS**
**Sintilimab** + **chemotherapy vs. Placebo** + **chemotherapy**	
Before two-stage correction	0.843
After two-stage correction	0.561
**Pembrolizumab** + **chemotherapy vs. Placebo** + **chemotherapy**	
Before two-stage correction	0.710
After two-stage correction	0.590

HR, hazard ratio; PFS, progression-free survival; OS, overall survival; MAIC, matching-adjusted indirect comparison.

Since the efficiency of placebo plus chemotherapy was evaluated in both ORIENT-12 and KEYNOTE-407, the anchored matching adjusted indirect comparison (MAIC) method (14) was adopted in this model. The PFS and OS data of the sintilimab group were chosen as the reference treatment to fit the pembrolizumab group using MAIC-adjusted HR (sintilimab group vs. pembrolizumab group). In the adjustment process, six key baseline demographic and disease characteristics factors, namely average age, gender, brain metastasis, stage of cancer, smoking history, and Eastern Cooperative Oncology Group (ECOG) score, which were reported in KEYOTE-407, were included. The results of baseline characteristics and adjusted HR are shown in Tables 2, 3.

**Table 2:** The result of baseline adjustment.

**Adjustment factor**	**Sintilimab group (before adjustment)**	**Pembrolizumab group**	**Sintilimab group (after adjustment)**
Proportion of male	91.60%	81.40%	81.40%
Average age (years)	61.48	65.00	65.00
Proportion of brain metastasis	3.92%	7.69%	7.69%
Proportion of stage IV cancer	65.83%	63.15%	63.15%
Proportion of smoking history	84.59%	92.67%	92.67%
Proportion of ECOG score = 1	85.43%	73.70%	73.70%

**Table 3:** HRs for PFS and OS after MAIC.

**Adjustment results**	**HR-PFS**	**HR-OS**
**Sintilimab + chemotherapy vs. Placebo + chemotherapy**		
Before MAIC	0.529	0.561
After MAIC	0.647	0.555
**Pembrolizumab + chemotherapy vs. Placebo + chemotherapy**	0.570	0.590
**Sintilimab + chemotherapy vs. Pembrolizumab + chemotherapy**	0.88	1.06

HR, hazard ratio; PFS, progression-free survival; OS, overall survival; MAIC, matching-adjusted indirect comparison.

Besides, due to the limited follow-up time in clinical trials, in order to obtain lifetime clinical data of patients, 6 types of parametric distribution models were used to extrapolate the lifetime survival outcomes of the sintilimab group based on the IPD of ORIENT-12. Akaike information criterion (AIC) and Bayesian information criterion (BIC) goodness-of-fit statistics along with visual inspection were used to evaluate optimal parametric distributions. As a result, the best-fitting distribution for PFS and OS data of sintilimab plus chemotherapy was log-normal and Weibull distribution, respectively. However, the visual inspection result was not good and there were logic errors in the Cholseky decomposition under the Weibull distribution, so the suboptimal distribution and log-normal distribution were chosen for OS data. Testing results were shown in the Supplementary material. The pembrolizumab group chose the same parametric distribution as the sintilimab group.

#### Utility weights

The utility values of health states were derived from the published literature (15) (PF = 0.804, PP = 0.321) (16), which used the time trade-off method to obtain metastatic NSCLC utilities in several countries, including China. In addition, the disutilities associated with the incidence of AEs with incidence ≥ 5% and grade ≥ 3 in the ORIENT-12 and KEYNOTE-407 studies were obtained from published literature (17, 18).

#### Resource use and costs

Patients in the state of PF were assumed to have drug costs, follow-up costs, administration costs, and management costs of AEs; patients in the state of PP, medical management costs and subsequent treatment costs were included. Drug costs included the cost of sintilimab, pembrolizumab, chemotherapy drugs, and subsequent treatments. The patient assistance plans (PAP) of sintilimab (19) and pembrolizumab (15) were taken into consideration. Subsequent treatments were aligned with the data of ORIENT-12, and the weighted costs were calculated by the proportion of different treatment options (shown in the Supplementary material). We assumed that the subsequent treatments of the pembrolizumab group were the same as that of the sintilimab group.

The cost of follow-up and medical service costs were calculated in two stages, namely, PF state and PP state. The unit price of each item of follow-up and medical service costs was obtained from the medical service price document of 11 provinces in China. Details of the calculation are shown in the Supplementary material.

Costs of AE management were estimated according to the duration of AEs and the incidence of AEs. AEs with an incidence ≥5% and grade ≥3 in the ORIENT-12 and KEYNOTE-407 studies were included in our study. The unit price of AEs treatment drugs was calculated based on the online price database (MEENET). The end-of-life care costs were derived from the published literature. In addition, we assumed that the mean weight of patients was 65 kg and the mean body surface area was 1.6 m^2^ to estimate the dosages of drugs, according to the recommendation from the National Healthcare Security Administration (NMPA) in China. All costs are expressed in 2021 US dollars (US$1 = 6.44 CNY). Details of all cost parameters are shown in Table 4.

**Table 4:** Key parameters and their variations.

**Parameters**	**Deterministic**	**Distribution**	**Low**	**High**	**Source**
**Unit drug costs ($)**					
Sintilimab	441.69	Constant	353.36	441.69	MENETxref ^*^
Pembrolizumab	2783.78	Constant	2227.02	2783.78	MENETxref ^*^
Paclitaxel (High dose)	26.26	Gamma	21.00	31.40	MENETxref ^*^
Paclitaxel (Low dose)	11.02	Gamma	10.56	11.47	MENETxref ^*^
Carboplatin (High dose)	8.04	Gamma	4.72	8.37	MENETxref ^*^
Carboplatin (Low dose)	4.72	Gamma	3.77	5.66	MENETxref ^*^
Gemcitabine	9.32	Gamma	1.24	9.94	MENETxref ^*^
Cisplatin (Low dose)	1.86	Gamma	1.13	2.96	MENETxref ^*^
Cisplatin (High dose)	2.66	Gamma	1.18	6.80	MENETxref ^*^
Subsequent treatment	4351.23	Constant	3480.98	5221.47	MENETxref ^*^
End-of-life care	2,298.86	Gamma	892.71	6,140.16	(20)
**Unit follow-up costs ($)**					
Imaging examination	57.48	Gamma	45.99	68.98	Health care document^**^
Blood chemistry	46.50	Gamma	37.20	55.80	Health care document^**^
Blood routine	3.11	Gamma	2.49	3.73	Health care document^**^
Urine routine	0.62	Gamma	0.50	0.75	Health care document^**^
**Unit medical service costs ($)**					
Diagnosis	3.11	Gamma	1.55	4.66	Health care document^**^
Intravenous injection	1.71	Gamma	1.55	2.14	Health care document^**^
Nursing	3.73	Gamma	2.98	4.47	Health care document^**^
Hospitalization	6.53	Gamma	5.22	7.83	Health care document^**^
**Unit AE management costs ($)**					
Neutrophil count decreased	115.01	Gamma	51.11	357.80	Expert opinion
White blood cell count decreased	115.01	Gamma	51.11	357.80	Expert opinion
Platelet count decreased	1,505.92	Gamma	1,240.17	1,771.67	Expert opinion
Anemia	138.75	Gamma	106.73	160.10	Expert opinion
**Incidence of AEs**					
**Sintilimab Arm**					
Neutrophil count decreased	15.1%	Beta	12%	18%	ORIENT-12 IPD
White blood cell count decreased	11.7%	Beta	9%	14%	ORIENT-12 IPD
Anemia	12.8%	Beta	10%	15%	ORIENT-12 IPD
Platelet count decreased	13.4%	Beta	11%	16%	ORIENT-12 IPD
**Pembrolizumab Arm**					
Neutrophil count decreased	23%	Beta	18%	28%	KEYNOTE-407
Platelet count decreased	8.3%	Beta	7%	10%	KEYNOTE-407
Anemia	15.8%	Beta	13%	19%	KEYNOTE-407
**Duration of AEs (Days)**					
Neutrophil count decreased	4.19	Normal	3.35	5.03	Expert opinion
Anemia	6.83	Normal	5.46	8.20	Expert opinion
White blood cell count decreased	4.5	Normal	3.60	5.40	Expert opinion
Platelet count decreased	47.29	Normal	37.83	56.75	Expert opinion
**Utilities**					
PF state	0.804	Beta	0.643	0.965	(16)
PP state	0.321	Beta	0.257	0.385	(16)
**Disutilities**					
Neutrophil count decreased	0.20	Beta	0.16	0.24	(16)
White blood cell count decreased	0.20	Beta	0.16	0.24	(16)
Platelet count decreased	0.11	Beta	0.09	0.13	(17)
Anemia	0.07	Beta	0.06	0.09	(18)

PF, progression-free; PP, post-progressive; IPD, individual patient data.

* The price of the drug was obtained from MENET, the online price database in China. (https://menet.com.cn).

** The price of follow-up and drug administration were obtained from the healthcare document of 11 provinces in China.

^***^The inclusion criteria of AEs were that the incidence of AEs ≥ 5% and grade ≥ 3.

### Sensitivity analyses

To verify the stability of model results, the one-way deterministic sensitivity analysis (DSA) and probabilistic sensitivity analysis (PSA) were performed. In the DSA, key parameters were varied by the standard error, 95% confidence interval, or ±20% of the deterministic value, except for the price of sintilimab and pembrolizumab (varied from 50 to 100%). PSA was performed using a second-order Monte Carlo simulation with 10,000 iterations. The parametric distribution assumptions were based on the recommended guidelines in *Decision Modeling for Health Economic Evaluation*. In addition, the survival parameters in the PSA were assessed through Cholesky decomposition.

### Scenario analysis

Since the literature utility values used in the base-case analysis were not fully applicable to Chinese patients with squamous NSCLC, the utility calculated based on the Research and Treatment Quality of Life Questionnaire-Core 30 (EORTC QLQ-C30) score collected from ORIENT-12 was used in the scenario analysis. According to the *NICE DSU Technical Support Document 10*, the mapping should be considered a second-best solution to collect EQ-5D values. We converted QLQ-C30 scores into EuroQol-5-dimension (EQ-5D) 5-level scores by adopting a mapping algorithm derived from published research (16).

**Figure 2:**
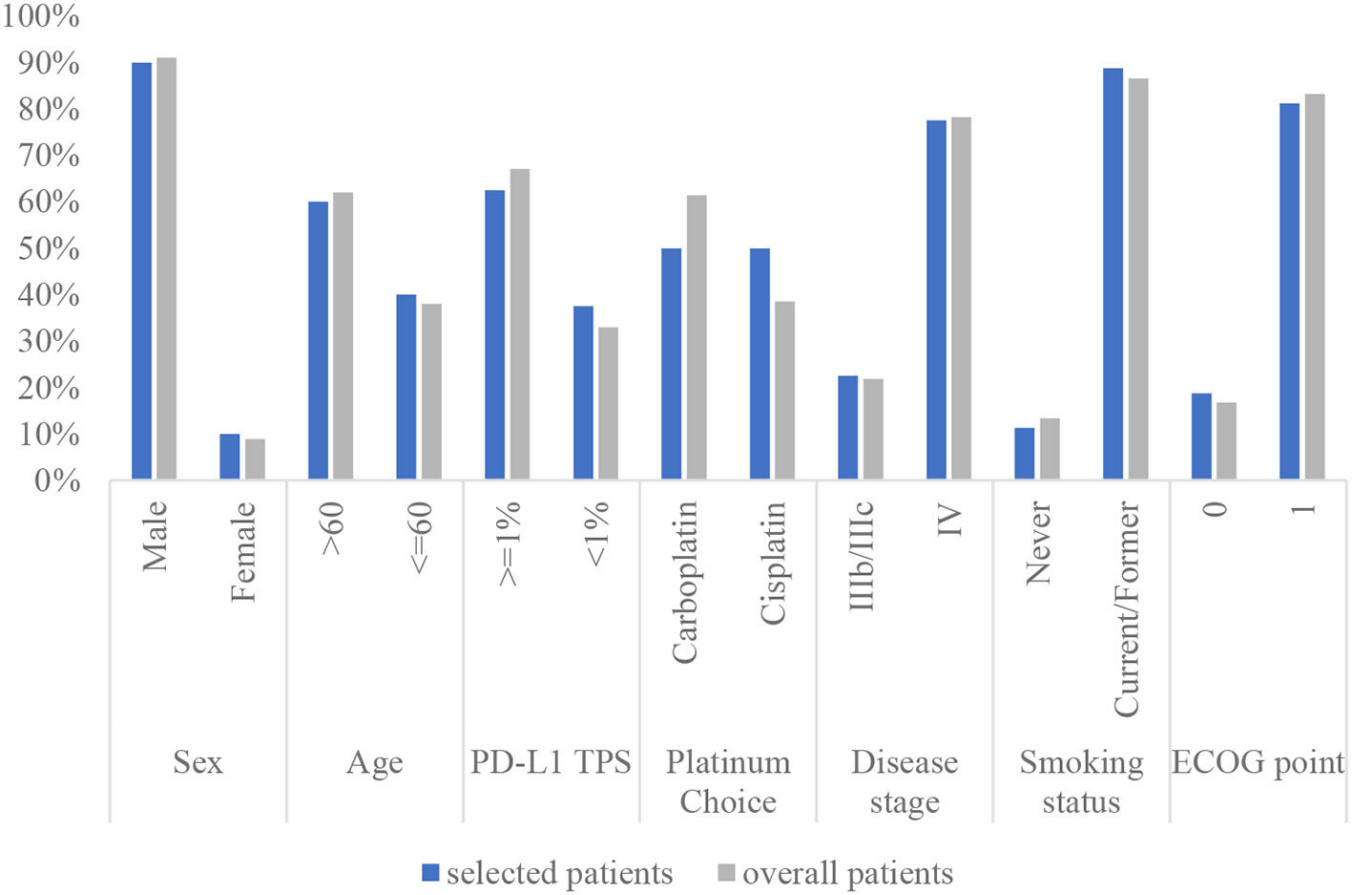
The characteristics of the selected patients compared with that of the overall patients in the clinical trial. PD-L1 TPS, programmed cell death-1 tumor proportion score; ECOG PS, Eastern Cooperative Oncology Group performance status.

To calculate the health state utilities, 80 patients were included after removing the logically incorrect data (health utilities of PF lower than those of PP). The baseline characteristics of the selected patients and all populations in the clinical trial are shown in Figure 2. For the sintilimab group, the utility value of PF and PP states was 0.730 and 0.615, respectively. Considering the utilities derived from patient-level data have included the impact of AEs, the health state utilities of the pembrolizumab group were adjusted according to the differential incidence of AEs between the two groups.

## Results

### Base case

The result of the base-case analysis is presented in Table 5. For Chinese advanced or metastatic squamous NSCLC patients, compared with pembrolizumab, the sintilimab strategy yielded lower QALYs of 0.0183 (0.9902 vs. 1.0085) and lower costs of $24,050 ($12,321 vs. 36,371). The ICER was $1,341,208/QALY, which indicated that a sintilimab strategy is a cost-effectiveness option under the WTP of 1–3 times the GDP per capita in China ($11,250/QALY~33,749/QALY).

**Table 5:** Summary of the cost and health outcomes results.

	**Sintilimab plus chemotherapy**	**Pembrolizumab plus chemotherapy**
QALYs	0.99	1.00
PF health state	0.65	0.73
PP health state	0.33	0.27
LYs	1.84	1.74
Total costs	$12,321	$36,371
Drug costs	$2,523	$26,768
Administration costs	$1,932	$1,866
Disease management and monitoring costs	$1,224	$1,162
AE costs	$250	$173
Subsequent therapy costs	$4,351	$4,351
End-of-life care costs	$2,039	$2,051
Incremental costs	-$24,050	
Incremental QALYs	−0.0183	
Incremental LYs	0.1005	
ICUR	$1,314,280/QALY	
ICER	Dominated	

QALY, quality-adjusted life year; PF, progression-free; PP, post-progressive; LY, life year; AE, adverse event; ICUR, incremental cost-utility ratio; ICER, incremental cost-effectiveness ratio.

### Sensitivity analyses

#### Deterministic sensitivity analyses

**Figure 3:**
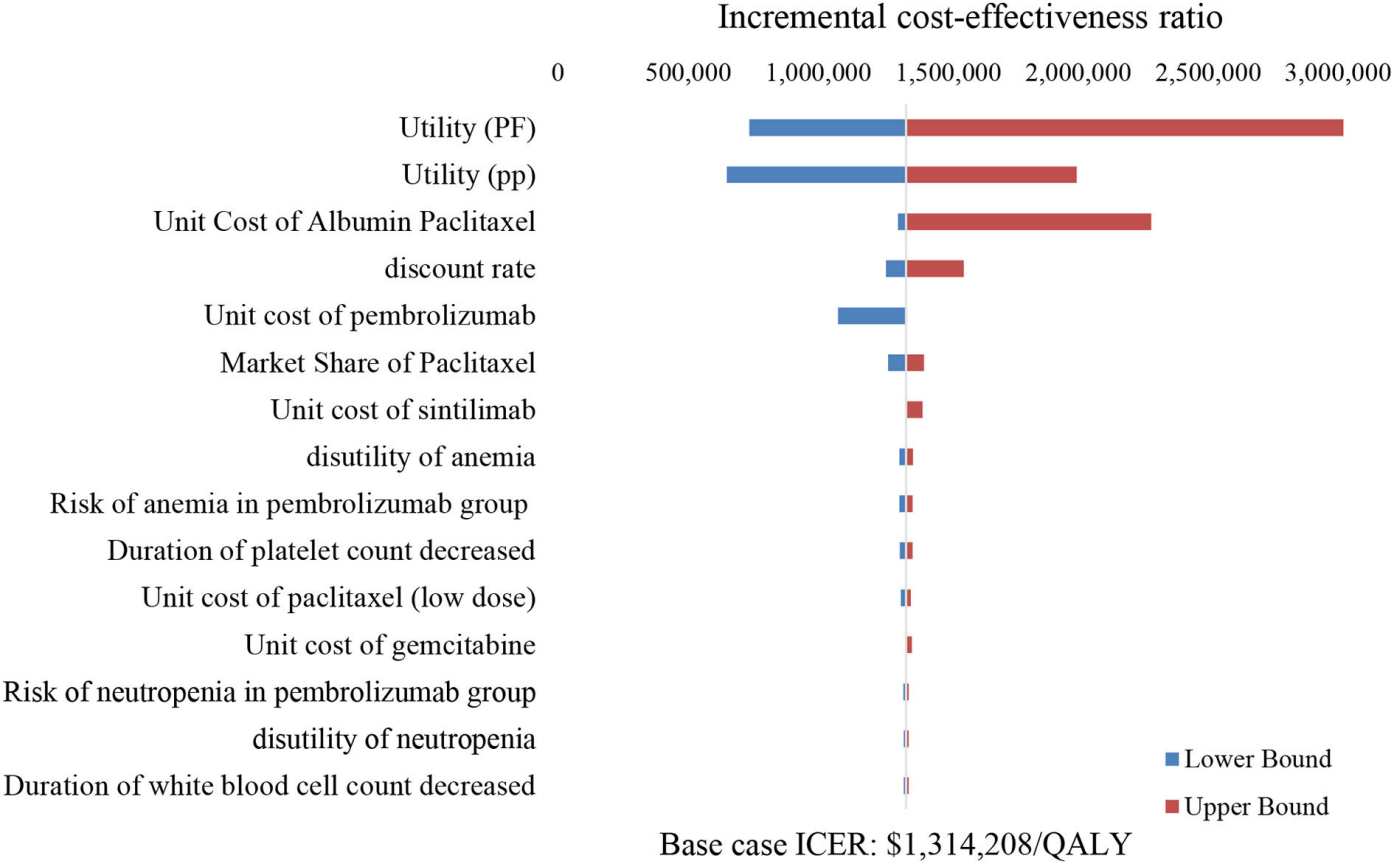
Tornado diagram. PF, progression-free; PP, post-progression.

The tornado diagram illustrated the top ten most influential key parameters in the one-way DSA (Figure 3). The utility of the PP state, the unit cost of albumin paclitaxel, and the utility of the PF state were the main driving parameters in the model, while other parameters had weak influences on the model results. As shown in Figure 3, the ICER value was most sensitive to the utility of the PP state, which implied that the changes in PP utility value may lead to a change in optimal strategy choice.

#### Probabilistic sensitivity analyses

**Figure 4:**
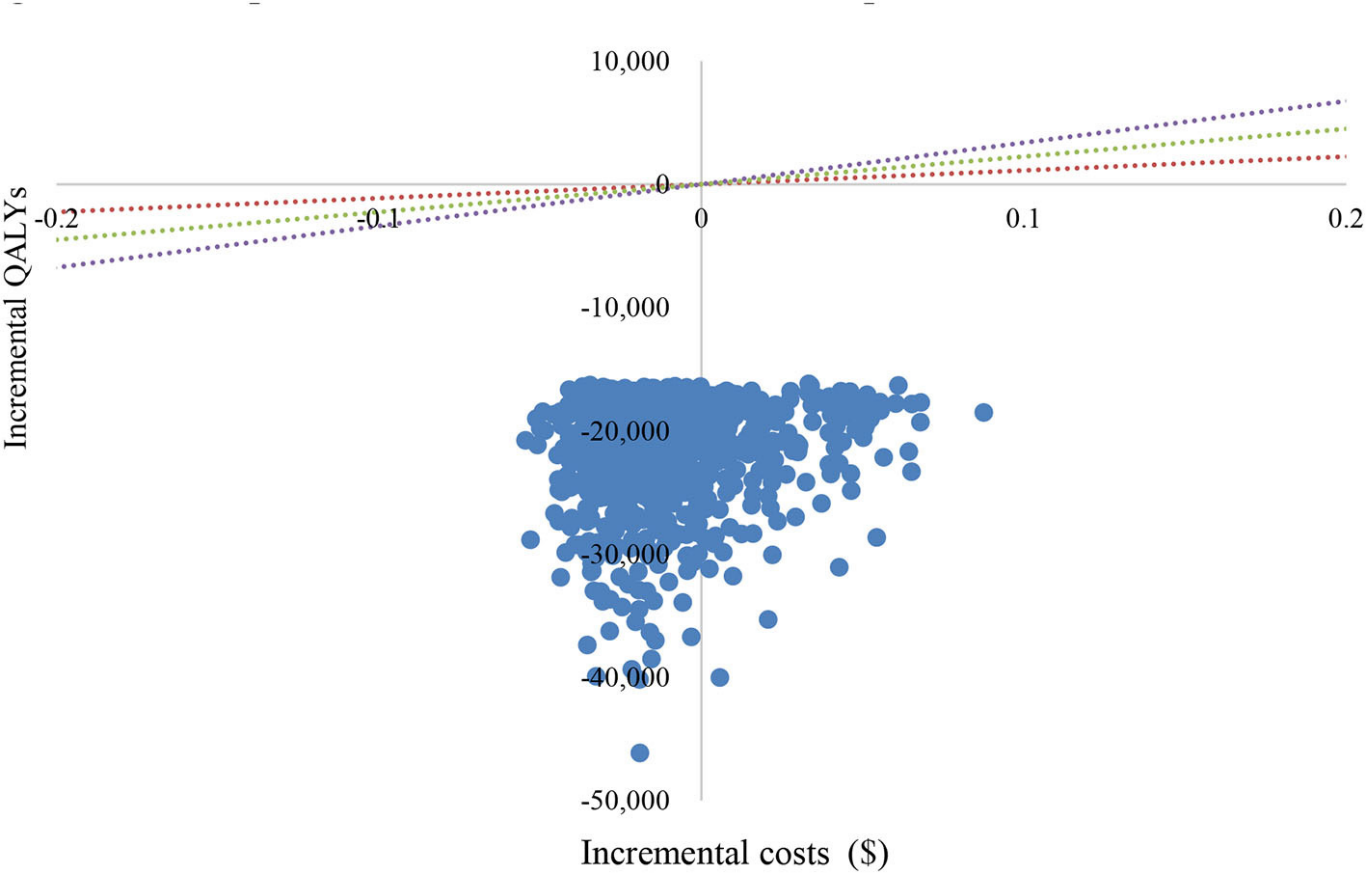
Scatter plot of incremental cost-effectiveness plane. QALY, quality-adjusted life year.

**Figure 5:**
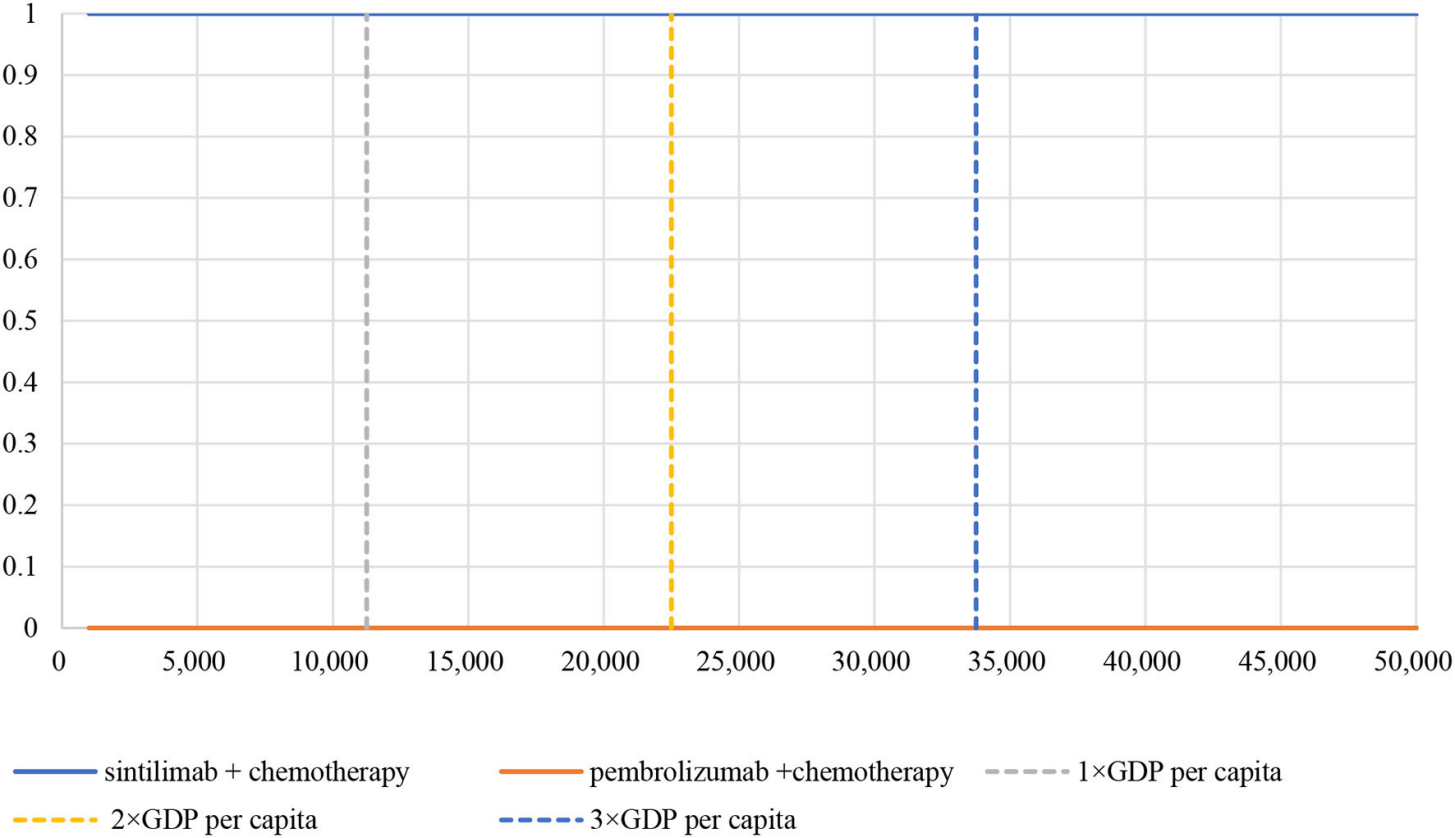
Cost-effectiveness acceptability curve. WTP, willingness-to-pay; GDP, gross domestic product.

The PSA showed an average QALY gain of −0.0168 and incremental costs of –$21,827, resulting in a probabilistic ICER of $1,299,226/QALY, which was consistent with the base-case results. A scatter plot of the incremental cost-effectiveness plane showed that most of the iteration results from the PSA fall in the third quadrant, while a small number fall in the fourth quadrant (Figure 4). According to the CEAC curve, at a WTP threshold of $11,250/QALY~$33,749/QALY (1–3 GDP per capita in China), the probability that sintilimab plus chemotherapy was cost-effective compared with pembrolizumab plus chemotherapy was almost 100% (Figure 5).

#### Scenario analysis

The scenario analysis results are shown in Table 6. Over a lifetime, the sintilimab plus chemotherapy group gained 1.23 QALYs with a cost of $12,321, while the pembrolizumab plus chemotherapy group gained 1.18 QALYs with a cost of $36,371. Compared with pembrolizumab plus chemotherapy, the incremental QALYs and cost for the sintilimab plus chemotherapy group were 0.0504 QALYs and –$24,050, which implied that sintilimab plus chemotherapy dominated pembrolizumab plus chemotherapy for the treatment of first-line advanced or metastatic squamous NSCLC in China. This was mainly because sintilimab plus chemotherapy obtained more QALYs during the PP state and the difference between PF and PP used in the scenario analysis was much smaller than that in the base-case analysis.

**Table 6:** The results of scenario analysis.

	**Sintilimab plus chemotherapy**	**Pembrolizumab plus chemotherapy**
QALYs	1.23	1.18
PF health state	0.59	0.67
PP health state	0.64	0.51
LYs	1.84	1.74
Total costs	$12,321	$36,371
Drug costs	$2,523	$26,768
Administration costs	$1,932	$1,866
Disease management and monitoring costs	$1,224	$1,162
AE costs	$250	$173
Subsequent therapy costs	$4,351	$4,351
End-of-life care costs	$2,039	$2,051
Incremental costs	-$24,050	
Incremental QALYs	0.0504	
Incremental LYs	0.1005	
ICUR	Dominated	
ICER	Dominated	

QALY, quality-adjusted life year; PF, progression-free; PP, post-progressive; LY, life year; AE, adverse event; ICUR, incremental cost utility ratio; ICER, incremental cost-effectiveness ratio.

## Discussion

The research evaluated the cost-effectiveness of sintilimab plus chemotherapy vs. pembrolizumab plus chemotherapy in patients with locally advanced and metastatic squamous NSCLC from a Chinese healthcare system perspective based on ORIENT-12 and KEYNOTE-407 studies.

Under the recommended thresholds of China's GDP per capita in 2020, the base-case results implied that sintilimab plus chemotherapy was more cost-effective vs. pembrolizumab plus chemotherapy. The result of PSA was in line with the base-case result, which shows that the sintilimab strategy has a high probability to be cost-effective. However, since the health outcome gap between the two strategies is very small, a small change in parameter value can cause a change in the study result. As shown in the scenario analysis and DSA, the result was sensitive to changes in utility value, which implied that the cost-effectiveness between these two strategies was not robust. Based on the breakdown results of QALYs, the benefits of the health outcome of sintilimab were mainly obtained in the PP stage. The difference in the incidence of AEs between the two strategies is the main reason for this phenomenon. Since the incidence of AEs in sintilimab is higher than pembrolizumab, the loss of health outcomes due to AEs in the PFS stage is higher. In addition, this also shows that patients treated with sintilimab will have a certain degree of improvement in quality of life even if their disease progresses.

The published economic evaluations of advanced NSCLC in China mainly compared pembrolizumab with chemotherapy (21, 22). However, although pembrolizumab shows good efficacy and safety for advanced NSCLC patients, it is usually not a cost-effective option in the Chinese context due to its expensive price. Recently, the listing of domestic PD-1 inhibitors, which have good cost performance, has provided more medication options for Chinese NSCLC patients. But there is a lack of economic evidence focused on domestic PD-1 inhibitors. To the best of our knowledge, this is the first study to compare the cost-effectiveness of sintilimab plus chemotherapy vs. pembrolizumab plus chemotherapy for NSCLC.

In addition, our study is important and instructive because it draws attention to some issues that should be heeded in the cost-effectiveness analyses of anti-oncology drugs when using indirect comparison methods, especially the crossover problems that can be solved by the two-stage method. Clinical trials for advanced cancers often adopted a crossover design. It means that patients are allowed to receive alternative therapy following disease progression on assigned treatment, which leads to a bias in the clinical efficacy of anti-oncology drugs. Cost-effectiveness analyses of oncology drugs usually obtain outcomes from crossover trials (23). Due to the crossover design, the treatment effect compared with the comparator on survival (such as HR) may be confounded (24). In this study, the two-stage method was used to adjust for the effect of subsequent-line therapies on survival outcomes for both the sintilimab and pembrolizumab groups to reduce the bias.

It should also be addressed that there were several limitations. First, the utility value used in both base-case and scenario analysis has limitations. For the utility value of base-case analysis, the target population for calculating this utility value includes not only squamous patients but also non-squamous patients, which did not exactly match the target population of this study. For the utility value of scenario analysis, only 80 patients were included in the utility value calculation, and the mapping formula is based on the UK population rather than the Chinese population, which might cause a bias in health outcomes. Second, the study relaxed the PH assumption of PFS and OS curves in the sintilimab group. PH hypothesis testing is supposed to be done in order to ensure curves used in the study meet the PH assumption when anchored indirect comparisons are applied. In the -ln(-ln(survival)) chart of sintilimab OS and PFS, the two curves remain parallel for most of the time with only a small overlap at the beginning of the curves. Therefore, the PH assumption was still assumed to be met in this study. Besides, although the best-fitting distribution for OS data of sintilimab plus chemotherapy was the Weibull distribution according to the results of AIC and BIC, the log-normal distribution was chosen because the visual inspection result was not good and there are many logic errors in the Cholesky decomposition under the Weibull distribution.

## Conclusion

According to the results of the base-case analysis and the sensitivity analyses, the QALYs gained between the sintilimab and pembrolizumab groups were similar, while the cost of the sintilimab group was much lower. Consequently, sintilimab plus chemotherapy is more cost-effective compared with pembrolizumab plus chemotherapy in China as the first-line treatment for locally advanced or metastatic squamous NSCLC patients in China.

## Data availability statement

The original contributions presented in the study are included in the article/Supplementary material, further inquiries can be directed to the corresponding author.

## Author contributions

PC, XW, SZ, MR, and YW developed the economic model and performed the analyses. XW and SZ interpreted the results and wrote the draft manuscript. XW, SZ, MR, and YW reviewed, analyzed, and interpreted the data. PC, HL, and AM contributed to the design of the primary model and the interpretation of the results. All authors reviewed and approved the final version.

## Funding

This study was supported by Innovent Biologics (Suzhou) Co., Ltd., Suzhou, Jiangsu, People's Republic of China. The authors were responsible for all content and editorial decisions, and they received no honoraria related to the development of this publication.

## Conflict of interest

HS was employed by Innovent Biologics (Suzhou) Co., Ltd. The remaining authors declare that the research was conducted in the absence of any commercial or financial relationships that could be construed as a potential conflict of interest.

## Publisher's note

All claims expressed in this article are solely those of the authors and do not necessarily represent those of their affiliated organizations, or those of the publisher, the editors and the reviewers. Any product that may be evaluated in this article, or claim that may be made by its manufacturer, is not guaranteed or endorsed by the publisher.
