# Supplementary material for: Economic evaluation of sintilimab plus chemotherapy vs. pembrolizumab plus chemotherapy for the treatment of first-line advanced or metastatic squamous NSCLC
Source: Front Public Health. 2022 Aug 9;10:956792. doi: 10.3389/fpubh.2022.956792 (PMC9395965; doi:10.3389/fpubh.2022.956792)
Supplement: Supplementary file 1 [file Data_Sheet_1.docx]

**Supplementary Material**

Figure S1 Parameter distribution of sintilimab+chemotherapy PFS


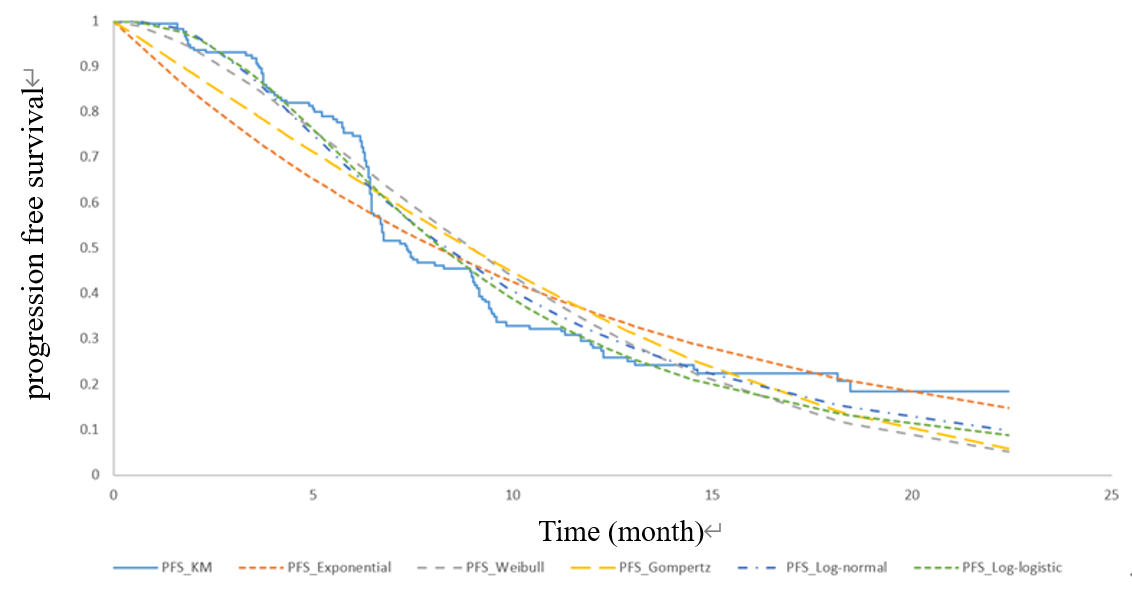


Figure S2 Parameter distribution of sintilimab+chemotherapy OS


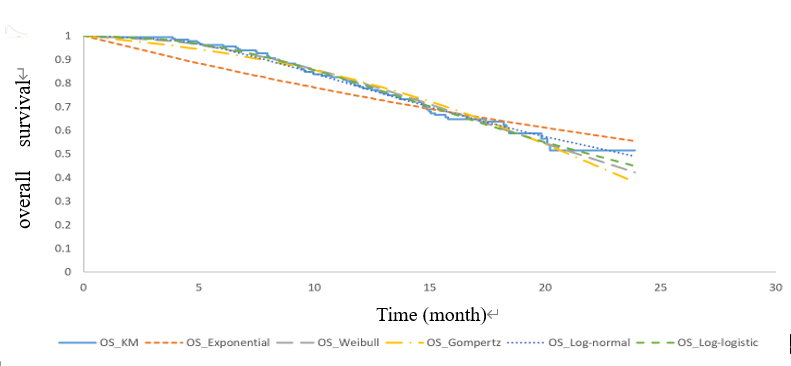


Table S1 Statistical testing of sintilimab +chemotherapy PFS

|  | Sintilimab+chemotherapy | |
| --- | --- | --- |
| Statistical testing of PFS | AIC | BIC |
| EXP | 423.5298 | 426.7171 |
| WEIBULL | 392.2924 | 398.6672 |
| GOMPERTZ | 414.4967 | 420.8714 |
| LOGNORMAL | 376.53 | 382.9047 |
| LOGGISTIC | 378.4515 | 387.8262 |

Table S2 Statistical testing of sintilimab +chemotherapy OS

|  | Sintilimab+chemotherapy | |
| --- | --- | --- |
| Statistical testing of OS | AIC | BIC |
| EXP | 312.0158 | 315.2032 |
| WEIBULL | 284.7094 | 291.0841 |
| GOMPERTZ | 291.7219 | 298.0967 |
| LOGNORMAL | 287.6195 | 293.9943 |
| LOGGISTIC | 287.9076 | 294.2823 |

Table S3 Subsequent treatment (only treatments for > 1% of patients were presented)

| Subsequent treatment | Proportion | Treatment duration (cycles) |
| --- | --- | --- |
| Docetaxel | 17.90% | 2.96 |
| Anlotinib | 14.50% | 2.75 |
| Paclitaxel | 14.00% | 2.88 |
| Recombinant human endostatin injection | 6.10% | 2.27 |
| Afatinib | 6.10% | 32.4 |
| Carboplatin | 5.60% | 3.78 |
| Nedaplatin | 2.80% | 2.4 |
| Cisplatin | 2.20% | 3 |

Table S4 Details of follow-up costs

|  |  | Frequency (a cycle) | | |
| --- | --- | --- | --- | --- |
| item | unit price ($) | 1-16 cycles  in PF state | after 16th cycles  in PF state | PP state |
| Imaging examination | 57.48 | 0.5 | 0.25 | 0.25 |
| Blood chemistry | 46.5 | 1 | 1 | 0.75 |
| Blood routine | 3.11 | 1 | 1 | 0.75 |
| Urine routine | 0.62 | 1 | 1 | 0.75 |
| cycle price ($) |  |  |  |  |
| 1-16 cycles in PF state | 78.97 | | | |
| after 16th cycels in PF state | 64.6 | | | |
| PP state | 52.04 | | | |

Table S5 Details of medical service costs

|  |  | Frequency (a cycle) | |
| --- | --- | --- | --- |
| item | unit price ($) | PF state | PD state |
| Diagnosis | 3.11 | 1 | 1 |
| Intravenous Injection | 1.71 | 3 | 3 |
| Nursing | 3.73 | 3 | 3 |
| hospitalization | 6.53 | 3 | 2 |
| cycle price ($) |  |  |  |
| PF state | 39 | | |
| PD state | 37 | | |
